# Bacterial phylogenetic tree construction based on genomic translation stop signals

**DOI:** 10.1186/2042-5783-2-6

**Published:** 2012-05-31

**Authors:** Lijing Xu, Jimmy Kuo, Jong-Kang Liu, Tit-Yee Wong

**Affiliations:** 1 Department of Biological Sciences, Bioinformatics Program, The University of Memphis, Memphis, TN, USA; 2 Department of Planning and Research, National Museum of Marine Biology and Aquarium, Pingtung, Taiwan; 3 Department of Biological Sciences, National Sun Yat-sen University, Kaohsiung, Taiwan

## Abstract

**Background:**

The efficiencies of the stop codons TAA, TAG, and TGA in protein synthesis termination are not the same. These variations could allow many genes to be regulated. There are many similar nucleotide trimers found on the second and third reading-frames of a gene. They are called premature stop codons (PSC). Like stop codons, the PSC in bacterial genomes are also highly bias in terms of their quantities and qualities on the genes. Phylogenetically related species often share a similar PSC profile. We want to know whether the selective forces that influence the stop codons and the PSC usage biases in a genome are related. We also wish to know how strong these trimers in a genome are related to the natural history of the bacterium. Knowing these relations may provide better knowledge in the phylogeny of bacteria

**Results:**

A 16SrRNA-alignment tree of 19 well-studied α-, β- and γ-Proteobacteria Type species is used as standard reference for bacterial phylogeny. The genomes of sixty-one bacteria, belonging to the α-, β- and γ-Proteobacteria subphyla, are used for this study. The stop codons and PSC are collectively termed “Translation Stop Signals” (TSS). A gene is represented by nine scalars corresponding to the numbers of counts of TAA, TAG, and TGA on each of the three reading-frames of that gene. “Translation Stop Signals Ratio” (TSSR) is the ratio between the TSS counts. Four types of TSSR are investigated. The TSSR-1, TSSR-2 and TSSR-3 are each a 3-scalar series corresponding respectively to the average ratio of TAA: TAG: TGA on the first, second, and third reading-frames of all genes in a genome. The Genomic-TSSR is a 9-scalar series representing the ratio of distribution of all TSS on the three reading-frames of all genes in a genome. Results show that bacteria grouped by their similarities based on TSSR-1, TSSR-2, or TSSR-3 values could only partially resolve the phylogeny of the species. However, grouping bacteria based on thier Genomic-TSSR values resulted in clusters of bacteria identical to those bacterial clusters of the reference tree. Unlike the 16SrRNA method, the Genomic-TSSR tree is also able to separate closely related species/strains at high resolution. Species and strains separated by the Genomic-TSSR grouping method are often in good agreement with those classified by other taxonomic methods. Correspondence analysis of individual genes shows that most genes in a bacterial genome share a similar TSSR value. However, within a chromosome, the Genic-TSSR values of genes near the replication origin region (Ori) are more similar to each other than those genes near the terminus region (Ter).

**Conclusion:**

The translation stop signals on the three reading-frames of the genes on a bacterial genome are interrelated, possibly due to frequent off-frame recombination facilitated by translational-associated recombination (TSR). However, TSR may not occur randomly in a bacterial chromosome. Genes near the Ori region are often highly expressed and a bacterium always maintains multiple copies of Ori. Frequent collisions between DNA- polymerase and RNA-polymerase would create many DNA strand-breaks on the genes; whereas DNA strand-break induced homologues-recombination is more likely to take place between genes with similar sequence. Thus, localized recombination could explain why the TSSR of genes near the Ori region are more similar to each other. The quantity and quality of these TSS in a genome strongly reflect the natural history of a bacterium. We propose that the Genomic- TSSR can be used as a subjective biomarker to represent the phyletic status of a bacterium.

## Background

The organization of genome is not random. Many of its features are correlated with abiotic and biotic stresses faced by individual species [1]. Stresses, such as translational selection, G+C pressure, GC skew between the leading and lagging strand, amino acid conservation, protein hydropathy, gene length, transcriptional selection, and the structural stability of RNA, often left behind many distinctive signatures on the genomes [2]. Among these features are various patterns of SNP [3], INDEL, [4], synonymous codons bias [5], codonpairs bias [6], and dipeptides bias [7]. Knowing these features have contributed significantly in our knowledge on molecular evolution, species phylogeny, and biotechnology [2,8]. We are interested in the organization of a lesser-known bias in the genomes – Translation Stop Signals (TSS), which is a collective term to describe the TAA, TAG, and TGA trimers on each of the three reading-frames of a protein coding genes.

TSS on the first reading-frame of the genes are called stop codons. Correct termination of protein synthesis is an important aspect of translational fidelity. Whereas sense-codons are recognized directly by base paring with the anticodons of tRNAs, the decoding of stop codons is mediated by proteins. In bacteria, a tripeptide in the bacterial release factors (RF) 1 and 2 serves as the “anticodon” in deciphering stop codons in mRNA. RF-1 recognizes UAA and UAG sequence in the mRNA, and RF-2 recognizes UGA and UAG in the mRNA. Furthermore, the efficiency and accuracy in terminating protein synthesis by UAA, UAG and UGA are not the same [9,10]. This flexibility of protein termination allows many genes to be regulated [11,12]. Since a stop codon acts on a single gene, and since genes within a genome are often diverse, the idea of using stop codon variations in a genome as biomarker for phylogenetic study has not been considered seriously.

There are also many off-frame “stop codons” on a gene. Off-frame stop codons are also called hidden stop codon, embedded stop codon, or premature stop codon (PSC) [13]. PSC may serve an essential function for the cell by preventing the ribosomes from misreading a gene [14,15]. Tse and associates have shown that the PSC-forming codon pairs are overrepresented in most of the 990 bacterial genomes they surveyed [16]. We have previously shown that the ratios of TAA: TAG: TGA in the genomes of phylogenetically related species are often similar [17]. In that same report, we also showed that species relatedness could not be constructed by comparing the ratios of three randomly picked nucleotide trimers. Also, the ratios of TAA: TAG: TGA on non-protein coding genes (such as tRNA, rRNA), or non-genic DNA (such as complimentary DNA sequences) does not exhibit phylogenic relatedness. Since the efficiency of protein termination by TAA, TAG, and TGA are quite different, we theorized that the type of PSC and the number of PSC on the genes of bacterial genomes are likely related to environmental adaptation and natural selection. For example, symbiotic bacteria (*EscherichiaFusobacterium, Rickettsia,* and *Borrelia*) would employ a “Many and Tight” strategy by having high number of PSC (> 80 per average gene) on their genes, and most of these PSC are of the error-proof type (TAA). Genes embedded with many error-proof TSS would effectively prevent new genes from forming via recombination. This “Many and Tight” strategy may benefit the symbionts because accidental formation of a protein of unknown function could interfere the normal symbiotic relation with the host. Whereas free-living bacteria and metabolically versatile bacteria, such as *Deinococcus, Mycobacterium, Pseudomonas*, and *Streptococcus* would use a “Few and Loose” strategy by having a few PSC (< 25 per average gene) on their genomes, and most of these PSC are of the error- prone type (TGA). For example, the number of PSC on the genes of *Staphylococcus aureus* is quite low. This versatile pathogen, which is well known for its resistance to antibiotics, is commonly found on the skin. Unlike the intracellular parasites, the environment of the skin changed rapidly. Having fewer PSC and using the error-prone type of TSS would increase of chance of creating new proteins with very different amino acid compositions rapidly via off-frame recombination. In turn, the new proteins might enhance the survival of the bacterium.

Since the quality and quantity of PSC in a genome could affect the fitness of a species [14,16,17], like the stop codons, PSC are likely subject to Darwinian selection. However, there are two different types of PSC. The TSS on the second reading-frame (i.e. N*TA-A*NN, N*TA-G*NN, and N*TG-A*NN) are formed by codon pairs where the lead codon contributes its last two nucleotides to the signal. There are only a few codons that can become the lead codon for the second reading-frame PSC, and they are all related to four nonpolar amino acids (L, I, V, M). On the other hand, the TSS on the third reading-frame are formed by codon pairs where the lead codons are all thymine-ending codons (NN*T-AA*N, NN*T-AG*N, and NN*T-GA*N). Most amino acids, except K, M, Q, E and G, have at least one thymine-ending synonymous codon. Thus, the contexts of PSC on the second and third frames are quite different. The formation of TSS on the second and third reading-frames might be subject to very different selective forces.

The interrelation between the stop codons and the PSC in a genome has never been investigated. In this communication, we wish to demonstrate that all the TSS in a bacterial genome are interrelated. Together, the ratio of these TSS of a genome could represent the phyletic status of a species. A mechanism is proposed to explain how TSS are populated in a bacterial genome. Understanding the role of TSS could provide further insight on the mechanism of genome evolution in bacteria.

## Results

### Comparing the TSSR-1 tree with reference tree

**Figure 1:**
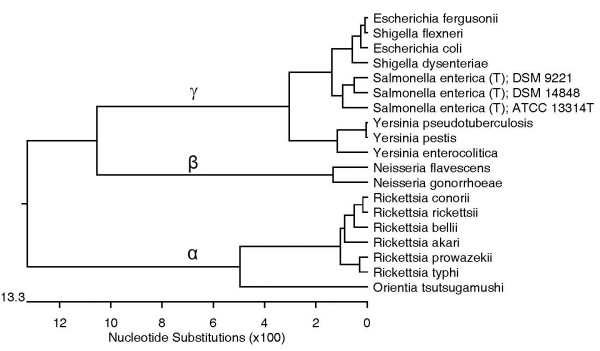
**Species correlations based on reading-frame-specific translation stop signals.** Hierarchical clustering of 61 bacteria (**A**) correlation based on the genomic translation stop signals ratios on the first reading frames (TSSR-1); (**B**) correlation based on the genomic translation stop signals ratios on the second reading frames (TSSR-2) and, (**C**) correlation based on the genomic translation stop signals ratios on third reading frames (TSSR-3). Correlation distance is between zero and one with zero being 100% similar, and one being no correlation.

**Figure 2:**
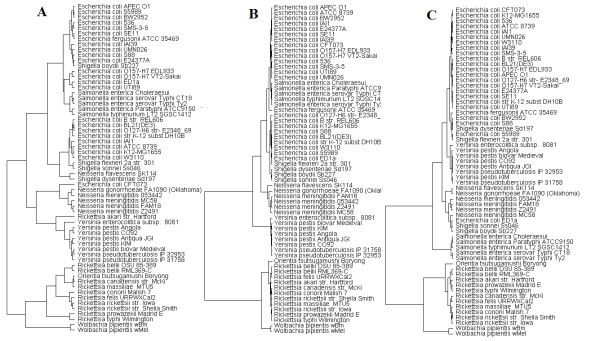
**16S rRNA alignment reference tree.** A phylogenetic reference tree is constructed from the 16SrRNA sequence alignment with 19 type species (see Table 1). This standard tree was used to validate the accuracy of other trees using bacterial translation stop signals profiles.

Hierarchical clustering techniques commonly used in DNA microarray studies [18], were used to correlate the distances between the TSSR values. A species is represented by the average value of its stop codons ratio (TSSR-1). A dendrogram showing the correlation between 61 bacterial genomes based on their TSSR-1is presented in Figure 1A. Bacteria belonging to the same genus often share a similar ratio of their stop codons usage. However, bacterial grouping based on TSSR-1 does not always agree with the reference tree (Figure 2). For example, *Yersinia* species and *Escherichia-Salmonella* group are all γ-Proteobacteria, but they are separated into two different branches on the TSSR-1 tree. The TSSR-1 tree also fails to resolve the distinction between *Escherichia* and *Salmonella* genera. Additionally, genotypic variations within a group can affect the TSSR-1 grouping significantly. Many individuals, such as the *Neisseria flavescens* SK114, *E. coli* CFT073, and *Rickettsia akari*, are not associated with their respective genera.

### Comparing the TSSR-2 tree with the reference tree

A species is represented by the average value of TSSR on the second reading-frames (TSSR-2). A dendrogram showing the distance correlation between the TSSR-2 of 61 bacteria is presented in Figure 1B. Bacteria grouped by their TSSR-2 are more cohesive. All bacteria belonging to the *Escherichia-Shigella-Salmonella* clade are grouped into a highly condensed cluster with two branches. The overall placement of bacteria on the TSSR-2 tree mimics that of the reference tree (Figure 2). However, like TSSR-1, the TSSR-2 tree fails to resolve the distinction between *Escherichia* and *Salmonella*, and the *Yersinia* group is separated from rest of the γ-Proteobacteria.

### Comparing the TSSR-3 tree with the reference tree

A species is represented by the average value of its TSSR on the third reading-frames (TSSR-3). The correlation of 61 bacterial TSSR-3 is shown in Figure 1C. The genera of *Yersinia* and *Escherichia-Shigella* are grouped but the genus *Salmonella* is separated from the other γ-Proteobacteria. In addition, *E. coli* ED1a, *Shigella sonnei* SS046, and *S. boydii* Sb227 are misplaced.

Bacteria correlations based on TSSR-1, TSSR-2, and TSSR-3 (Figure 1A–C) have provided different clues on their relatedness. Nevertheless, none of them alone could accurately place all the test organisms to their correct phyletic position (Figure 2).

### Comparing the genomic-TSSR tree with the reference tree

**Figure 3:**
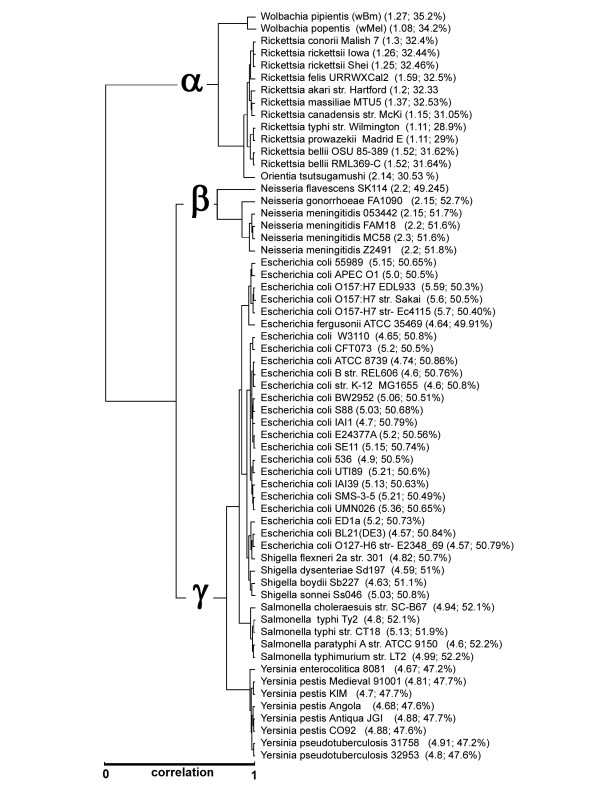
**Species correlation based on genomic translation stop signals on all three reading-frames.** Distance correlation of 61 bacteria based on their Genomic Translation Stop Signals Ratio. A species is represented by the average value of all its Genic-TSSR (Genomic-TSSR). The Genomic-TSSR values of 61 bacterial genomes were clustered by Hierarchical clustering by City-Block Distance, Complete-Linage. Parentheses show the genomic size and GC ratio of that species. Correlation distance is between zero and one with zero being 100% similar, and one being no correlation.

A different tree is produced when each bacterium is represented by the average value of all its Genic-TSSR (Genomic-TSSR) (Figure 3). The branches and members on the branches of this tree are in complete agreement with those on the reference tree (Figure 2). Additionally, species and subspecies are clustered with very high resolution. A detail description of this tree is described:

Organisms on the first branch are all members of the α-Proteobacteria*.* This branch has three sub-branches: *Rickettsia* (11 species/strains), *Orientia* (1 species), and *Wolbachia* (2 species). Genomic-TSSR grouping of these bacteria is not influenced by genomic sizes or by their GC contents. The genomic size of bacterium in this branch varies from 1.08 Mb to 2.14 Mb, and their GC content ranges from 28.9 to 35.2%.

The tree generated by Genomic-TSSR values also exhibits very high resolution. All 11 *Rickettsia* species/strains are clustered into one group with two distinct terminals separating the typhus causing bacteria (*R. prowazekii* and *R. typhi*) and the spotted-fever causing bacteria. The two *R. bellii* strains are more closely related to the typhus causing bacteria. The Genomic-TSSR distinction between the Typhus and Spotted subgroup is in good agreement with the current scheme of Rickettsial classification [19]. *Orientia tsutsugamushi* is the out-group of the *Rickettsia*. This Genomic-TSSR assignment of *Rickettsia-Orientia* is in perfect agreement with the reference tree (Figure 2) and is supported by many other independent evidences [19].

*Wolbachia* (2 strains) forms a outer cluster of the *Rickettsiae* group. Although not well characterized, we included *Wolbachia* in this study solely for in the hope to get new information that could resolve the phyletic status of this interesting bacterium. Filariasis is a leading cause of global disability. Most of these filarial nematodes are dependent on a symbiosis with *Wolbachia* bacteria [20]. Strains assignment for *Wolbachia* is problematic. As to the date of this writing, the Ribosome Data Project Database has yet to assign a type 16SrRNA sequence to represent *Wolbachia*. However, there are multiple lines of evidences to suggest a close genetic association between *Wolbachia* and *Rickettsiae*[20-22]. Currently, *Wolbachia* has only one species – *W. pipientis*. The insect-harbored *W. pipientis* wMel and the round worm–harbored *W. pipientis* wBm, differ in host specificity and GC content (34.2% vs. 35.2%). Despite these differences, the Genomic-TSSR correlation between these two stains of *Wolbachia* is very close. The *Wolbachia* Genomic-TSSR is also closely associated the Genomic-TSSR values of other α-Proteobacteria.

Members on the second branch of the Genomic-TSSR tree are all β- Proteobacteria. This branch includes 6 species/strains of *Neisseria.* Most *Neisseria* are commensal. Detailed subgrouping of *Neisseria* is often problematic [23]. Unlike the *RickettsiaNeisseria* are often considered sexual because they are naturally competent [24]. The degree of genetic relatedness between *N. gonorrhoeae* and *N. meningitidis* is extremely high [25]. Despite such high degree of genetic similarity, the Genomic-TSSR values of the four strains of *N. meningitidis* form a tight group separated from the *N. gonorrhoeae*. The Genomic-TSSR correlation among *Neisseria* species is in perfect agreement with that of the 16SrRNA sequence alignment tree (Figure 2).

Members of the third branch of the Genomic-TSSR tree are all γ-*Proteobacteria*. The genomic size of individual organisms in this group varies from 5.7 Mb to 4.6 Mb, and the GC content varies from 51.2 to 47%. Within this branch are two distinct sub-branches: The *Escherichia*-*Salmonella* sub-branch and the *Yersinia* sub-branch. The *Escherichia-Salmonella* sub-branch has 43 genera: *E. coli* (23 strains) and *E. fergusonii*, *Shigella* (4 species), and *Salmonella* (5 species), whereas the *Yersinia* sub-branch has 8 species/strains.

Most microbiologists believe *Shigella* is a clone of *E. coli*[26]. Without any exception, all the 28 *Escherichia-Shigella* species/strains are clustered into a tight group. This strongly suggests that the Genomic-TSSR value is not influenced by genome variation of individual strains. All *Salmonella* are also grouped as a single clade. Within the *S enterica* strains, the four human pathogens form a tight sub-group separated from the swine isolate, *S. choleraesuis*. Although highly correlated, the Genomic-TSSR values of the typhoid-fever strains are separated from the Genomic-TSSR values of the paratyphoid-fever strains. The Genomic-TSSR tree showing *Salmonella* is the next-of-kin to the *Escherichia-Shigella* is in perfect agreement with the 16SrRNA sequence alignment tree (Figure 2) and other independent evidences [27].

The Genomic-TSSR of *Yersinia* forms a distinct cluster separated from the *Escherichia-Shigella- Salmonella* group. Traditionally, *Y. pestis* can be separated into three major biovars – Antiqua, Orientals, and Medievalis. The Genomic-TSSRs of the Antiqua (*Y. pestis* Angola and *Y. pestis* Antiqua) and the Orientalis (*Y. pestis* CO92) biovars are very similar. The Genomic-TSSR of the Antiqua-Orientalis group and *Y. pseudotuberculosis* is also very close. However, the Medievalis strains (*Y. pestis* 91001 and *Y. pestis* KIM) form a cohesive branch outside the *Y. pseudotuberculosis* branch. *Y. enterocolitica* is the root of the *Yersinia* clade. In general, the Genomic-TSSR correlation scheme of this group of bacteria is in line with other phylogenetic scheme proposed [28]. However, some slight differences are noticed. Based on the sequences of five selected housekeeping genes, it was proposed that *Y. pestis* was evolved recently as a clone of *Y. pseudotuberculosis*[29]. Our study showed that Antiqua and Orientalis are likely the decedents of *Y. pseudotuberculosis*. However, the Medievalis strains (*Y. pestis* 91001 and *Y. pestis* KIM) form a cohesive branch outside the *Y. pseudotuberculosis* branch. This might suggest multiple origins of *Y. pestis*. Thus, the Genomic-TSSR assignment for Medievalis phylogeny is inconsistent with the 5-housekeeping-genes assignment.

### TSSR variations of individual genes

**Figure 4:**
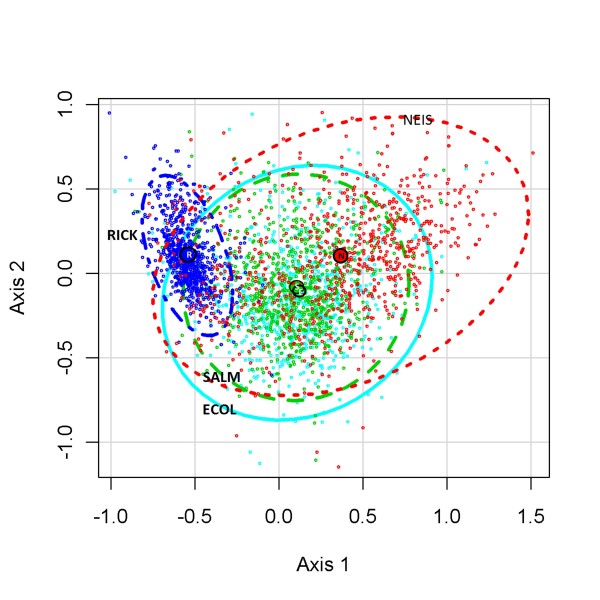
**Correspondence Analysis of individual genes from four different species.** Five hundred randomly selected genes from each of the genomes of four different bacteria were selected for CA analysis. At 95% confidence, four clusters of genes could be recognized: *Escherichia coli* CFT073 (ECOL, solid line, Turquoise), *Salmonella typhimurium* LTS (SALM, dashed line, Green), *Rickettsia typhi* Wilmington (RICK, dotted-dashed line, Dark Blue) and *Neisseria meningitidis* Mc58 (NEIS, dotted line, Red). Also showed are the centroids of the genes of *E. coli* (E), *S. typhimurium* (S), R. typhi (R), and *N. meningitidis* (N).

The Genomic-TSSR is the average value of all Genic-TSSR on the genome of that bacterium. However, the average value could be skewed by a few dominating genes. To investigate this issue, five hundred genes from each of four different bacterial genomes were randomly selected. Their Genic-TSSR relations were analyzed using the CA technique. CA is a statistical method able to analyze and plot a cloud of values of multiple dimensions (in our case, there are nine dimensions, representing the each of the nine TSS), and rotate it so that the maximum variability is visible. The distributions of 2000 Genic-TSSR values from four different genomes are presented in Figure 4. At 95% confidence, four clusters of genes are recognized. The *Escherichia* (ECOL, solid line, Turquoise) and *Salmonella* (SALM, dashed lines, Green) genes form two concentric ellipses with most of their genes overlapping the same space. The centroids of *Escherichia* (E) (coordinates = +0.11, -0.08) and *Salmonell*a (M) (coordinates = +0.10, -0.11) are very close to each other. The *Neisseria* genes (NEIS, dotted line, Red) are wider spread. The centroid of the *Neisseria* genes (N) is located at the upper right side of the graph (coordinates = +0.37, +0.11). The cloud of the Rickettsial genes (RICK, dotted-dashed line, Dark Blue) is highly condensed. The centroid of the rickettsial genes (R) is located at the upper left side of the graph (coordinates = −0.53, +0.10).

Figure 4 suggests that most of the genes in a species share a similar Genic-TSSR value and that the TSSR of the genes of related species, such as *Escherichia* and *Salmonella*, are very similar. The coordinates of the centroid, which is essentially the average value of the genes, of *Escherichia* and *Salmonella* are very close. This directly supports the Hierarchical distance clustering result of Figure 3.

### TSSR bias of individual genes within a chromosome

**Figure 5:**
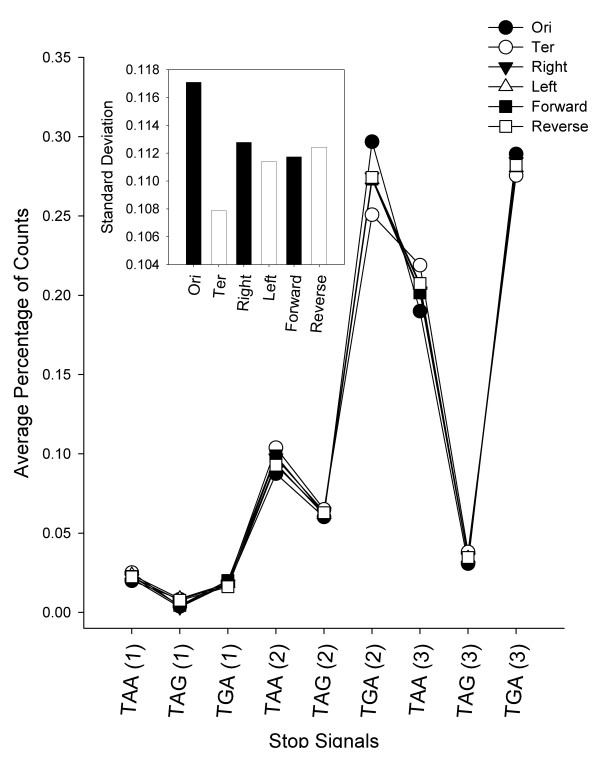
**Kolmogorov-Smirnov test for discrete distributions of Genic-TSSRs on a chromosome.** A two-sided Kolmogorov-Smirnov test (KS-test) comparing the average percentage of counts of each translation stop signals among 400 genes from *Escherichia coli* K12 based on assigning these genes to three categories. (**A**) genes are assigned based on the location on each replichore (Left (∆) vs. Right (▼)); (**B**) genes are assigned based on their orientation on the leading or lagging stands of the DNA (Forward (■) vs. Reverse (□)), or (**C**) genes are assigned based on their proximity to the replication origin or terminus (Ori (●) vs. Ter (○)). The insert shows the standard deviation of the average percentage of counts of all nine types of translation stop signals in each gene assignment.

DNA replicates from the replication origin (Ori) to terminus (Ter). It divides a bacterial chromosome into oppositely replicated halves, which are referred as replichores. DNA sequences between the two replichores are often biased. The frequencies of occurrences of many short sequences, such as the Chi sites, on each of the replichores are very different [30]. Also, because of GC-skew and other factors, the orientation of genes on the leading and lagging strands of DNA [31,32], and genes locating near the Ori and Ter [33] are often biased. We wanted to know whether genes on the two replichores, the orientation of the genes on different DNA strands, or the proximities of the genes to the Ori and Ter, would affect the TSSR value of the genes. The genome of *E. coli* K12 was used to investigate this issue. Results (Figure 5) show that when genes are categorized based on their location on the left or right replichores, or based on the orientation on the leading or lagging strands of the DNA, the average percentage of TSSR were essentially the same (p = 1). However, when genes are categorized by their proximity to the Ori or Ter, the TSSR of the genes near Ori (●) and the TSSR of the genes near the Ter (○) regions are statistically different (p = 0.2). For *E. coli*, among the nine different TSSR scalars, the TGA signal on the second reading-frame (N*TGA*NN) is most distinct. The average percentage of counts for this signal in genes near the Ori is about 0.3, whereas the average percentage of counts for this signal in genes near the Ter region is only 0.25. The variations between TSSR usages among gene groups can be better visualized by plotting the standard deviations (SD) of the means of the nine TSSR of the above three pairs of data (Figure 5 insert). The SD of the genes on the left and right replichores are very similar (about 0.112 ±0.00055). Similarly, genes locating on the leading and lagging strains of the DNA do not show any significant different in their Genic-TSSR values. However, the SD of the TSSR in genes near the Ori region is higher (SD = 0.117088) is much higher whereas the SD of the TSSR in the genes near the Ter region is much lower (SD = 0.107883). Similar results were also observed in the genomes of *N. meningitidis* Z491 and *Yersinia pestis* Kim (data not shown). 

## Discussion

The 16SrRNA alignment tree is currently the primary reference for bacterial phylogeny [34]. This “gold standard” is often used by researchers to prove (or disprove) the phylogenic relation of species based on other biomarkers [35,36]. We employed a similar strategy to test the possible use of Genomic-TSSR as a tool for bacterial phylogeny. We also used multiple strains of the same species to provide a measure of genotypic variation within a species.

It has been known that stop codon usage is influenced by natural selection [37,38], but genomic bias in stop codons usage has never been considered as a valid biomarker for species identification. Failure in using genomic stop codons bias as an effective biomarker for species identification is clearly illustrated in Figure 1A. Although most phylogenetically related species can be grouped by their ratio of stop codons usages at lower taxons, genotypic variations within a species could misidentify a strain. Genomic stop codon bias also fails to predict the bacteria at higher taxon. *Yesinia* is placed apart from γ-*Proteobacteria* (Figure 1A).

Many reports also suggested that the TSS on the second and third reading-frames are subject to natural selection [14-17,39]. We found that related species often share a similar TSSR on their second or third reading-frames (Figure 1B1C). However, like the stop codon bias, neither TSSR-2 nor TSSR-3 alone can reliably predict the identity of a species (Figure 1 vs. Figure 2). Despite the different contexts of the TSS on each of the three reading-frames, there are certain features between the TSSR-1, TSSR-2, and TSSR-3 trees that seem to complement each other. We initially noticed that, by sorting the numeric values of one or other TSSR column on the spreadsheet (Table 2), one or other groups of phylogenetically related groups of bacteria (on the rows) would come closer together. We therefore decided to consider all nine signals simultaneously. Our initial thought was that, by providing certain weights on certain class of TSS, related species might form a cluster. Much to our surprise, the Genomic-TSSR correlation tree was in complete agreement with the 16SrRNA tree, without any mathematical manipulation (Figure 3 vs. Figure 2). This result not only suggests that the TSS on each of the three reading-frames in a genome are interrelated, the complete symmetry between the Genomic-TSSR and 16SrRNA trees suggests the Darwinian selection force on TSS in directing the evolution of Proteobacteria is parallel to that of the rRANs. Why are the TSSs on the three reading-frames of a genome interrelated to each other? A possible mechanism is proposed:

Most bacterial genes are formed by gene duplication, recombination and divergence [40]. Off-frame recombination would instantaneously generate a set of new sense-codons, which are important for rapid gene divergence. Unlike those sense-codons that dictate the physical character for a protein, TSS in the genes would dictate the length, and therefore the complexity, of future genes [17]. The DNA is the common template for both chromosome replication and gene transcription. In bacteria, both DNA replication and gene expression occur simultaneously. When a bacterium divides, the faster moving DNA replication machinery often collides with the slower moving transcription machinery on the same track of DNA. This would cause the supercoiled DNA between these two complexes to break, leading to recombination at that region. This phenomenon, termed transcription-associated recombination (TAR), has proven to be a major player in the maintenance of genome integrity and in the induction of genetic instability and diversity [41-43]. Off-frame recombination induced by TAR may explain why the TSS in a genome are interrelated: Frequent collisions between DNA and RNA polymerases would increase the frequency of homologous recombination at the protein-coding regions of the chromosome. Off-frame recombination would shuffle the TSS between the three reading-frames and from one gene to other genes. Repetitive TAR during the course of species evolution could explain why the TSS on the three reading-frames of the genes in a chromosome are interrelated, and why closely related species always share a similar Genomic-TSSR.

The GC content on the leading strands and lagging strands on the chromosome are skewed [44]. However, GC skew does not seem to affect the TSSR on the leading and lagging oriented genes. Genes on each replichore also share a similar TSSR profile (Figure 5). Perhaps, the intrinsic compositions of TSS, which are rich in A and T, poor in G, and lack of C, may avoid the bias of CG skew. Instead, we noticed that the TSSR of genes between the Ori and Ter regions are quite different (Figure 5). This regional bias may be related to the mechanism of bacterial chromosome replication. Initiation of DNA replication at Ori proceeds bidirectionally and terminates at the Ter region [45]. Very often, the rate of chromosome replication is slower than the rate of cell division. Bacterium compensates the slower DNA replication process by initiating multiple rounds of DNA replication before each cell division [46]. As a result, the copies of genes near the Ori region are amplified, a phenomenon commonly known as replication-associated gene dosage. For example, when *E. coli* are grown at rates of 2 doublings/h, genes near the Ori are about threefold more prevalent than genes near the terminus; even at a very slow growth rate of 0.6 doubling/h, this ratio is still significantly high (about 1.7) [47]. In addition, genes near the Ori region are often highly expressed genes [48]. Thus, the frequency of TAR induced homologues recombination among genes near the Ori region is expected to occur more often. As a result, the TSS on the genes near the Ori region would shuffle more often among themselves than the rest of the genes on the same chromosome. This could explain why the TSS in the genes near the Ori are more similar to each other. Similarly, the Ter region is the site of decatenation of circular chromosomes by topoisomerase IV [49]. Arrest of the replication fork near the Ter region often exposes a single-stranded gapped region and DNA ends from the newly replicated strands at the fork junction, which is subjected to homologous recombination near that region [50].

The Genomic-TSSR calculation is based on averaging the Genic-TSSR values of all genes in a genome. Horizontal gene transfer from unrelated species would undoubtedly disrupt the TSS profile of a species. There are some evidences to support this view: For examples, at the genome level, the Genomic-TSSR correlation between members of a genus that evolved without the input of foreign genes, such as the *Rickettsia* species [51], or those species that are evolved only recently, such as the *Yersinia* species [52], are very high (Figure 3). On the other hand, *Neisseria* are often considered promiscuous because they are naturally competence [53]. The Genomic-TSSR correlation between the *Neisseria* species is also less cohesive (Figure 3). Furthermore, at the individual gene level, the Genic-TSSR values of individual genes in the nonsexual *Rickettsia* genome are tightly clustered whereas the Genic-TSSR values of individual genes of the promiscuous *Neisseria genomeare* wider spread on the CA plot (Figure 4).

Classification of bacteria based on monophasic method such as rRNA sequence alignment [54] often lacks resolution [34]. A single measurement is also subject to simple stochastic variation and to the influence of horizontal gene transfer [55]. Parallel methods of classification based on multilocus sequences from selected species [56] are also problematic. The 1988 report from the Ad Hoc Committee on Reconciliation of Approaches to Bacterial Systemics urged caution about inferring phylogeny tree based on any one class of conserved molecules [57] and the 2002 Ad Hoc Committee for the Re-evaluation of the Species Definition in Bacteriology [58] recognized the importance of whole-genome in classification. Whole-genome approaches, based on large data base comparisons [59] and shared orthologous gene/biomarkers profiles often require subjective selection of phenotypic and molecular biomarkers [60,61]. The selections of biomarkers are sometimes controversial [61,62]. The rule(s) for picking a core set of genes, or defining a type species remained problematic [63]. More importantly, most of these methods utilized similar sequence alignment tools, such as BLAST, for grouping. Algorithms used to align and delineate DNA sequences could be bias [59,64]. Ideally, a bacterium should be represented by all the genes in its genome. The TAA, TAG, and TGA trimers are universally found in protein-coding genes. The novel method describes herein represents a robust, whole-genome, and theory-based solution for bacterial classification.

## Conclusion

The translation stop signals on the three reading-frames of the genes on a bacterial genome are interrelated, possibly due to frequent off-frame recombination facilitated by translational-associated recombination (TSR) coupled with the manner of bacterial DNA replication. We propose that the Genomic- TSSR can be used as a subjective biomarker to represent the phyletic status of a bacterium.

## Method

### Verification of phylogenic relation

Inference of bacterial phylogeny is based on the 16SrRNA alignment tree of 19 well-studied bacteria belonging to the subphyla of the-, β-, andγ-Proteobacteria. The ClustalW program in the DNA Star software (Lasergene, WI) was used to create a reference phylogenetic tree (Figure 2). Sixty-one genomes of well-characterized species belonging to the above subphyla were selected for testing.

### Data sources

Nineteen ‘good quality’, ‘type strain’ 16SrRNA sequences were downloaded from the Ribosomal Database Project server (http://rdp.cme.msu.edu) (Table 1). The FASTA nucleic acid files of 61 bacterial chromosomal genes were downloaded from the Comprehensive Microbial Research website (http://cmr.tigr.org). Except that of the *Wolbachia*, well-characterized and monophyletic bacterial groups were selected for this study to insure accuracy. Bacterial species and their Taxon ID are listed in Table 2. This table is also posted on our website (http://umdrive.memphis.edu/tywong/public/Table_1jb) in Excel format.

**Table 1:** List of reference type species

**GenBank sequence**	**Type Species; ID**
S000004313	*Escherichia coli* (T); ATCC 11775T
S000139289	*Escherichia fergusonii* (T); ATCC 35469
S000414306	*Neisseria flavescens* (T); L06168
S000003950	*Neisseria gonorrhoeae* (T); NCTC 83785
S000414651	*Rickettsia akari* (T); MK (Kaplan)
S000414655	*Rickettsia bellii* (T); 369L42-1
S000500280	*Rickettsia conorii* (T); Malish 7
S000436058	*Rickettsia prowazekii* (T); M21789
S000414667	*Rickettsia rickettsii* (T); L36217
S000437122	Rickettsia typhi (T); Wilmington;
S000006115	*Salmonella enterica* (T); ATCC 13314T
S000926440	*Salmonella enterica* (T); DSM 14848
S000926444	*Salmonella enterica* (T); DSM 9221
S000000258	*Shigella dysenteriae* (T); X96966
S000013935	*Shigella flexneri* (T); X96963
S000392501	*Yersinia enterocolitica* (T); ATCC 9610
S000392506	*Yersinia pestis* (T); NCTC 5923
S000392498	*Yersinia pseudotuberculosis* (T); ATCC 29833
S000413795	*Orientia tsutsugamushi Karp*; D38623

The 16SrRNA sequences of 18 type species from Ribosomal Database Project were used for the construction of reference phylogenetic tree in Figure 2.

**Table 2:** Organisms used in this study and their corresponding genomic Translation stop signal ratios (Genomic-TSSR)

**S**	**ID**	**Organism**	**1**	**2**	**3**	**4**	**5**	**6**	**7**	**8**	**9**
α	357244	Orientia tsutsugamushi Boryong	0.010	0.003	0.003	0.186	0.157	0.063	0.281	0.102	0.195
	293614	Rickettsia akari str. Hartford	0.009	0.004	0.005	0.200	0.164	0.061	0.277	0.088	0.192
	391896	Rickettsia bellii OSU 85-389	0.009	0.003	0.003	0.198	0.163	0.053	0.285	0.086	0.200
	336407	Rickettsia bellii RML369-C	0.009	0.003	0.002	0.198	0.164	0.053	0.286	0.086	0.199
	293613	Rickettsia canadensis str. McKi	0.010	0.003	0.003	0.205	0.163	0.057	0.280	0.090	0.189
	272944	Rickettsia conorii Malish 7	0.010	0.004	0.003	0.206	0.165	0.058	0.279	0.089	0.185
	315456	Rickettsia felis URRWXCal2	0.008	0.004	0.003	0.204	0.167	0.054	0.283	0.088	0.189
	416276	Rickettsia massiliae MTU5	0.008	0.003	0.002	0.206	0.166	0.056	0.281	0.091	0.186
	272947	Rickettsia prowazekii str. Madrid E	0.009	0.002	0.002	0.204	0.163	0.059	0.283	0.086	0.193
	452659	Rickettsia rickettsii str. Iowa	0.011	0.004	0.004	0.205	0.165	0.059	0.279	0.088	0.185
	392021	Rickettsia rickettsii str. Shei	0.010	0.004	0.004	0.204	0.167	0.058	0.279	0.089	0.186
	257363	Rickettsia typhi str. Wilmington	0.008	0.002	0.002	0.202	0.161	0.059	0.284	0.086	0.195
	80849	Wolbachia pipientis Drosophila (wBm)	0.016	0.009	0.008	0.198	0.153	0.098	0.209	0.082	0.228
	955	Wolbachia pipientis Brugia (wMel)	0.011	0.006	0.005	0.201	0.158	0.095	0.216	0.085	0.223
_β_	218491	Neisseria flavescens SK114	0.027	0.004	0.010	0.078	0.074	0.329	0.176	0.025	0.278
	242231	Neisseria gonorrhoeae FA1090	0.026	0.006	0.020	0.098	0.066	0.402	0.141	0.021	0.222
	374833	Neisseria meningitidis 053442	0.024	0.006	0.018	0.095	0.069	0.380	0.152	0.024	0.232
	272831	Neisseria meningitidis FAM18	0.023	0.005	0.019	0.096	0.072	0.381	0.152	0.025	0.227
	122586	Neisseria meningitidis MC58	0.024	0.006	0.018	0.097	0.073	0.372	0.157	0.026	0.227
	122587	Neisseria meningitidis Z2491	0.025	0.006	0.019	0.097	0.070	0.383	0.152	0.024	0.225
γ	316407	Escherichia coli W3110	0.020	0.003	0.012	0.091	0.064	0.274	0.205	0.040	0.291
	362663	Escherichia coli 536	0.018	0.002	0.009	0.092	0.066	0.272	0.207	0.040	0.296
	585055	Escherichia coli 55989	0.017	0.002	0.009	0.089	0.060	0.269	0.209	0.039	0.305
	405955	Escherichia coli APEC O1	0.017	0.002	0.008	0.092	0.064	0.270	0.209	0.039	0.301
	481805	Escherichia coli ATCC 8739	0.018	0.002	0.008	0.091	0.065	0.275	0.206	0.040	0.295
	413997	Escherichia coli B str. REL606	0.018	0.002	0.008	0.090	0.064	0.277	0.207	0.039	0.295
	469008	Escherichia coli BL21(DE3)	0.019	0.002	0.008	0.089	0.064	0.278	0.206	0.039	0.295
	574521	Escherichia coli BW2952	0.018	0.002	0.009	0.091	0.064	0.272	0.207	0.039	0.299
	199310	Escherichia coli CFT073	0.019	0.003	0.012	0.095	0.064	0.271	0.205	0.040	0.291
	331111	Escherichia coli E24377A	0.018	0.002	0.009	0.091	0.063	0.272	0.207	0.039	0.298
	585397	Escherichia coli ED1a	0.018	0.002	0.010	0.090	0.062	0.277	0.205	0.037	0.301
	585034	Escherichia coli IAI1	0.018	0.002	0.008	0.090	0.064	0.273	0.208	0.040	0.296
	585057	Escherichia coli IAI39	0.018	0.002	0.009	0.092	0.062	0.272	0.209	0.038	0.297
	574521	Escherichia coli O127-H6 str- E2348_69	0.018	0.002	0.008	0.088	0.064	0.279	0.205	0.039	0.295
	155864	Escherichia coli O157:H7 EDL933	0.017	0.002	0.010	0.093	0.062	0.267	0.209	0.040	0.300
	83334	Escherichia coli O157:H7 str. Sakai	0.017	0.002	0.010	0.093	0.062	0.268	0.208	0.039	0.300
	444450	Escherichia coli O157-H7 str- Ec4115	0.018	0.003	0.010	0.092	0.062	0.268	0.209	0.039	0.301
	585035	Escherichia coli S88	0.018	0.002	0.009	0.090	0.064	0.275	0.206	0.038	0.299
	409438	Escherichia coli SE11	0.018	0.002	0.009	0.091	0.063	0.273	0.207	0.039	0.298
	439855	Escherichia coli SMS-3-5	0.018	0.002	0.009	0.093	0.064	0.270	0.210	0.039	0.295
	168927	Escherichia coli str. K-12 MG1655	0.018	0.002	0.008	0.090	0.064	0.277	0.207	0.040	0.293
	585056	Escherichia coli UMN026	0.017	0.002	0.009	0.093	0.064	0.272	0.208	0.039	0.296
	364106	Escherichia coli UTI89	0.018	0.002	0.009	0.093	0.065	0.272	0.206	0.040	0.295
	585054	Escherichia fergusonii ATCC 35469	0.017	0.002	0.008	0.094	0.067	0.258	0.207	0.041	0.305
	209261	Salmonella typhi Ty2	0.018	0.003	0.010	0.091	0.068	0.273	0.217	0.045	0.275
	321314	Salmonella choleraesuis str. SC-B67	0.020	0.004	0.013	0.094	0.069	0.270	0.216	0.045	0.269
	295319	Salmonella paratyphi A str. ATCC 9150	0.018	0.003	0.009	0.091	0.069	0.272	0.219	0.046	0.272
	220341	Salmonella typhi str. CT18	0.018	0.003	0.010	0.091	0.068	0.272	0.217	0.045	0.276
	99287	Salmonella typhimurium str. LT2	0.018	0.003	0.009	0.092	0.068	0.272	0.219	0.045	0.274
	300268	Shigella boydii Sb227	0.021	0.004	0.014	0.088	0.060	0.280	0.201	0.036	0.297
	300267	Shigella dysenteriae Sd197	0.024	0.004	0.013	0.084	0.062	0.279	0.203	0.038	0.294
	198215	Shigella flexneri 2a str. 301	0.019	0.002	0.008	0.090	0.063	0.279	0.204	0.037	0.297
	300269	Shigella sonnei Ss046	0.020	0.003	0.011	0.088	0.061	0.283	0.202	0.035	0.297
	393305	Yersinia enterocolitica 8081	0.014	0.003	0.006	0.088	0.096	0.219	0.209	0.048	0.316
	349746	Yersinia pestis Angola	0.015	0.004	0.007	0.087	0.094	0.223	0.213	0.045	0.312
	360102	Yersinia pestis Antiqua JGI	0.014	0.003	0.007	0.088	0.094	0.223	0.213	0.046	0.312
	229193	Yersinia pestis biovar Medieval 91001	0.013	0.004	0.007	0.090	0.093	0.225	0.211	0.046	0.311
	214092	Yersinia pestis CO92	0.013	0.003	0.007	0.088	0.094	0.224	0.212	0.046	0.312
	187410	Yersinia pestis KIM	0.014	0.004	0.007	0.090	0.094	0.224	0.211	0.047	0.309
	349747	Yersinia pseudotuberculosis IP 31758	0.013	0.004	0.007	0.088	0.094	0.221	0.214	0.047	0.313
	273123	Yersinia pseudotuberculosis IP 32953	0.013	0.003	0.007	0.088	0.094	0.221	0.214	0.047	0.314

Proteobacteria strains used in this study are grouped in the order of α-, β-, and γ- subphyla(S). Their Taxon identifications (ID) and their corresponding Genomic Translation stop signals Ratios (TSSR) are listed. Columns labeled 1–9 represent the rations of TAA: TAG: TGA of the 1^st^, 2^nd^, and 3 reading-frames, respectively, of the Genomic Translation Stop Signal Ratios (TSSR) of that species. A more detailed table is posted on our website (http://umdrive.memphis.edu/tywong/public/Table_1jb).

### Classification of translation stop signals on a gene

A script written in C (downloadable at our website (https://umdrive.memphis.edu/tywong/public/codon_062107.zip) was used to count the frequencies of occurrences of TAA, TAG, and TGA on the 1^st^, 2^nd^, and 3^rd^ reading-frames on each and all genes in a genome. For example, consider the following hypothetical gene composed of 15 codons:

ATG, GTA, AGG, GTG, AGT, ATA, ATG, GTA, GCC, GGT, GGT, TAA

The script converted the above hypothetical gene into a 9-scalar TSS series separated into 9 columns:

1, 0, 0, 2, 1, 1, 

where the first 3 scalars represent the number of TAA (=1), TAG (=0) and TGA (=0) TSS on the first reading-frame of this gene. The second three scalars are the number of TAA (=2), TAG (=1) and TGA (=1) TSS (single-underline) on the second reading-frame of this gene. The third three scalars are the number of TAA (=1), TAG (=1), and TGA (=1) TSS (double-underline) on the third reading-frame of this gene.

The First reading-frame TSS ratio (TSSR-1) is referred as the ratio of TSS (TAA: TAG: TGA) on the first reading-frame (columns 1–3) of a gene. For the above hypothetical gene, the Genic-TSSR-1 value is 1, 0, 0. The Genomic-TSSR-1 is the average value of all Genic-TSSR-1 of a genome. The Second reading-frame TSS ratio (TSSR-2) is defined as the ratio of TSS on the second reading-frames (columns 4–6). For the above hypothetical gene, the Genic-TSSR-2 value is 0.5, 025, 0.25. The Genomic-TSSR-2 is the average value of all Genic-TSSR-2 of a genome. The Third reading-frame TSS ratio (TSSR-3) is defined as the ratio of TSS on the third reading-frame (columns 7–9) of a gene. For the above hypothetical gene, the Genic-TSSR-3 value is 0.33, 0.33, 0.33. The Genomic-TSSR-3 is the average value of all Genic-TSSR-3 of a genome. A Genic- TSS ratio (Genic-TSSR) is defined as the ratio of all nine scalars (columns 1–9) of a gene. For example, the Genic-TSSR value of the hypothetical gene is equal to 0.125, 0.00, 0.00, 0.25, 0.125, 0.125, 0.125, 0.125, 0.125. Genomic- TSS ratio (Genomic-TSSR) is defined as the average of Genic-TSSR of all genes in a genome. For example, the genome of *Escherichia coli* K12 consist of 4289 genes, The TAA, TAG, and TGA counts on the 1^st^, 2^nd^, and 3^rd^ reading-frames of all the genes (TSS) in this bacterium equal to 2707, 326,1256, 13755, 9780, 42155, 31463, 6048, and 44610, respectively. The sum of all TSS equals to 152100. The Genomic-TSSR for this bacterium is calculated by dividing each of the 9 scalars by the sum of all TSS, generating a TSSR series of 0.02, 0.00, 0.01, 0.09, 0.06, 0.28, 0.21, 0.04, 0.29 to represent *E. coli* K12 (See Table 2). In calculating the Genomic-TSSR, genes that have multiple reading-frames (such as those annotated as “authentic frameshift” genes) were deleted from the dataset.

### Hierarchical correlation analysis

The hierarchical clustering algorithm in Cluster 3.0was downloaded from Michael Eisen’s website (http://rana.lbl.gov/EisenSoftware.htm). The TSSR dendrogram was constructed using the Java TreeView software available from the Java TreeView website (http://jtreeview.sourceforge.net/). The scale of the tree was from zero to one, with zero meaning no correlation and 1 meaning 100% similar.

### Correspondence analysis (CA) of individual genes from four different species

We selected four bacteria, two of which are phylogenetically related (*Escherichia coli CFT073* and *Salmonella typhi TY2*), and two of which are phylogenetically unrelated (*Rickettsia typhi Wilmington and Neisseria meningitidis MC58*) to show the correlation between individual genes among these organisms. Five hundred genes were randomly selected from each bacterium. The 2000 genes were pooled and each of their Genic-TSSR value calculated. We treated each of the nine scalars on the Genic-TSSR as nine independent columns and each gene as an independent row for CA analysis. The R “ade4” package for CA analysis was downloaded from the R-project website (http://www.r-project.org). CA mapped the selected genes into a 9-dimensional space according to the nine scalars of the Genic-TSSRs. Then it plotted the major TSS as those axes through the multidimensional hyperspace that accounted for the largest fraction of the variation among genes. A list of the genes, together with their corresponding Genic-TSSR values is posted on our website (https://umdrive.memphis.edu/tywong/public/genic_TSSR).

### TSSR bias of individual genes within a chromosome

The genome of *Escherichia coli* K12 strain was used to test whether TSSR bias exist in a genome. One hundred genes from each of the left and right sides at the coordinate 3923499 were selected to represent the genes near the Ori region. Similarly, 100 genes from each of the left and right sides of the coordinate 1588799 were selected to represent the genes near the Ter region. The selected 400 genes were also grouped based on the orientation or based on their location on the left or right replichores. The gene names and their Genic-TSSR are listed on our website (https://umdrive.memphis.edu/tywong/public/OrivsTer).

### Kolmogorov-Smirnov test for discrete distributions of genic-TSSRs on a chromosome

The Kolmogorov-Smirnov test (KS-test) is a robust test that cares only about the relative distribution of the data (i.e. it is a non-parametric and distribution free method). The hypothesis regarding the distributional form is rejected if the test statistic, *D* (the observation values of KS-test) is greater than the critical value. The two-sided KS-test uses the maximum vertical deviation between the two curves (control vs. treatment) as the statistic *D* and provides a graphical presentation, which enables the user to detect normal distributions of the data. We sorted the 400 genes by three different categories. The first category was to assign the selected genes based on their location on the left or right replichore (Left vs. Right). The second category was to assign the genes based on their orientation on the leading or lagging strands of DNA (Forward vs. Reverse). The third category was to assign genes based on their proximity to the replication origin or terminus regions (Ori vs. Ter). We than calculated the average percentage of counts of the pair in each category by the two-sided KS-test. To insure data were not skewed by a few dominating genes, we perform 1000 random samplings (M = 1000). In each sampling, 200 genes were randomly selected twice. One set of data was assigned as control group and the other set of data was assigned as treatment group. The KS-test was performed and the *D* statistic obtained from sampling was used to compare with the *D* statistic generated from the gene assignment among each pair. The *p*-value was calculated as: $p=\frac{\sum_{m=1}^{M}I\left(D_{m}\geq D\right)}{M}\text{,}$ where I (.) is the indicator function. If the condition in parentheses is true, it equals to 1, else 0.

## Competing interest

The authors declare that they have no competing interests.

## Authors’ contributions

LIX performed the two-sided KS-test analysis and discussion, JK contributed the CA result and discussion, JKL participated in bacterial systematic and discussion, and TYW conceived and designed the experiment, carried out experiments and drafted the manuscript. All authors read and approved the final manuscript.
